# An Empirical Research on the Behavioral Perceptions of University Students on Their ERASMUS Mobilities Abroad

**DOI:** 10.3390/ijerph19095756

**Published:** 2022-05-09

**Authors:** Nicolae Marinescu, Anca Madar, Nicoleta Andreea Neacsu, Camelia Schiopu

**Affiliations:** 1 Department of Marketing, Tourism-Services and International Business, Faculty of Economic Sciences and Business Administration, Transilvania University of Brasov, 500036 Brasov, Romania; ancamadar@unitbv.ro (A.M.); andreea.neacsu@unitbv.ro (N.A.N.); 2 Department of Management and Economic Informatics, Faculty of Economic Sciences and Business Administration, Transilvania University of Brasov, 500036 Brasov, Romania; camelia.s@unitbv.ro

**Keywords:** university students, European Union, academic performance, quantitative research, ERASMUS mobilities

## Abstract

The European Union (EU) labor market needs a better-skilled workforce that can work in any of the Member States. In order to develop the skills and competencies of future competitors in this market, several student mobility programs have been set up in the EU, where they can travel to universities in partner countries to further their studies and enrich their academic performance, culture and knowledge. The best known of these programs is ERASMUS (European Region Action Scheme for the Mobility of University Students). Because Romania also participates in this program in the exchange of university students, the authors wanted, through this study, to highlight the benefits and challenges of participation in the program, to analyze how international mobilities are emotionally perceived by students and what are the behavioral reasons that determine Romanian students to choose a certain university as a study destination abroad. For this purpose, the authors conducted quantitative marketing research among students from the Transilvania University of Brasov who have been abroad with ERASMUS scholarships. The research results show that an important component in choosing a certain university is not the financial expense during the mobility as might have been expected, but rather the initial desire to study abroad. Students also consider the improvement of their academic performance as an equally important reason for embarking on mobility to the social aspect of getting to know other cultures.

## 1. Introduction

In recent years, the international mobility of students has become an important goal, whereby learners shape their formation in different environments, in a foreign language and benefit from a cross-cultural experience [1]. Today, society has become multicultural due to enhanced mobility and globalization. An increasing part of business is characterized by teamwork with great cultural, professional and individual diversity [2]. That is why there is a need for mobility to help students meet colleagues from other countries. Given that the labor market is liberalized in the European Union (EU), students need to be prepared to take up a job anywhere in the EU.

The ERASMUS program, established in 1987, represents a part of the European Commission’s initiatives in higher education. The program is partially funded by the European Union and is very popular with university students. The ERASMUS program is based on written collaboration agreements between universities, as stated by Derzsi et al., 2011 [3].

Given that Romania is also part of the ERASMUS student mobility program from the 1998–1999 academic year and that the flow of university students to and from Romania has steadily increased, the authors of this paper are entitled to identify the behavioral perceptions of Romanian students participating in this type of mobility, regarding the reasons why they chose a certain university, what emotional challenges they encountered during the mobility, how they adapted in the country where they left, etc.

Improving the mobility process is an important goal of the European Higher Education Area and a priority of the European Union for the modernization of higher education, society, as well as the improvement of educational policies. Mobility is also paramount for the professional development of university students, their academic performance and for enhancing their empathy toward international skills and cooperation.

This study analyzes the overall picture of the mobility of higher education students from an academic and socio-economic perspective. The paper focuses, in particular, on the factors that attracted university students to participate in such a program and on identifying the emotional challenges, behavioral problems and necessary resources encountered during mobility, trying to understand the costs of such mobility and how the academic performance in another international university is assessed in comparison to the home university [4].

The authors conducted an opinion poll among students at the Transilvania University of Brasov who benefited from ERASMUS mobilities in universities in the EU. The survey was conducted on the basis of a questionnaire, and a computer-assisted interviewing (CAWI) technique was used to collect the data. This technique implies displaying the questionnaire on a certain website, and, subsequently, respondents complete the answers to the questions in an online manner.

The results of the research show that monthly financial expenses during mobility are not an important component when choosing a university for a scholarship abroad. The cost of mobility influences the choice of the host university only in 12.5% of cases. However, a fairly high percentage (90.18%) of respondents stated that they had additional financial resources during their mobility. The main reason why university students choose ERASMUS mobility (regardless of gender and study cycle) is the desire to study abroad. They also opt for the mobility to enhance their academic performance in the same vein as getting to know other cultures. The reputation of the host university ranks significantly higher for Master’s students compared to Bachelor’s students. The major emotional challenge consists in coping with different teaching and examination methods, while the number-one success factor of the mobility (90% of cases) is considering the student–teacher relationship.

## 2. Literature Review

Cooperation to foster development in the field of university education has become possible on a larger scale with the enlargement of the European Union and the desire to achieve a strong educational area to compete with other regions of the world [5]. The enlargement of the European Union through the entry of new states has led to an increase in the flow of students between different universities [6]. Thus, a number of EU-financed programs have been set up to support university students in participating in such cultural and professional interactions and to deepen the impact of higher education and vocational education on the research and innovation process [7]. Their aim is to increase the qualification level of graduates so that they can face the competition in the labor market, competition determined by the phenomenon of globalization [8].

The need and importance of student mobilities arise from the requirements of the labor market toward high-skill jobs, the international competition for talent and the desire to curb youth unemployment. ERASMUS mobilities are perceived to enhance the knowledge, skills and academic performance of university students, thereby raising employability and responsible citizenship. As a consequence, they became one of the central elements of the European Commission’s strategies in the area of education.

As observed in the Berlin Communique (2003), student mobilities embrace several different real-life dimensions: political, social, economic, as well as academic and cultural ones [9].

During the years of implementation of the Bologna Process, recent times brought a shift in the way the value of mobility was assessed, with increased attention awarded to the importance of mobility for employability. Thus, it was considered that benefits reach beyond academics and culture to foster positive developments in the European labor market [10].

Indeed, EU officials considered mobility to be a tool for combating youth unemployment, one of the major goals of the Europe 2020 strategy for growth and jobs. A 2014 study demonstrated that the danger of lasting structural unemployment decreased substantially for students who undertook mobility compared to those who did not take the opportunity to study abroad [11].

In the literature, mobilities are classified according to several criteria, as stated by Skribnjek [12].

According to the country where students come from EU universities, there are: vertical mobilities—those mobilities in which university students come from outside the EU for a higher level of education, and horizontal mobilities—intra-European mobilities, between programs of equal value.

According to the duration spent abroad, there are: degree mobilities—the duration is until obtaining a university degree, and credit mobilities—the duration is necessary to continue studies, but their completion (obtaining a university degree) is achieved in the country of origin.

According to the direction of the flow of university students, there are: incoming mobilities—when foreign students come to a university in a particular country, and outgoing mobilities—referring to students leaving home to go to universities in other countries.

**Figure 1:**
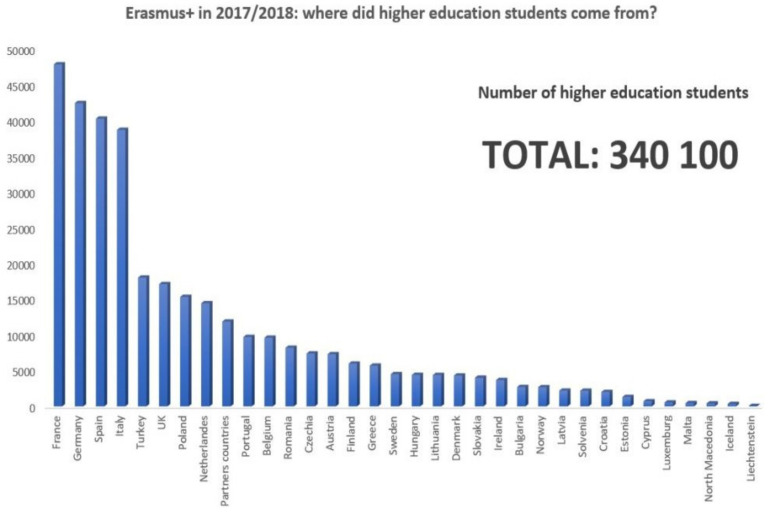
Home countries of university students with ERASMUS+ mobilities. *Source: ERASMUS Annual Report 2018,* *European Commission* [13].

**Figure 2:**
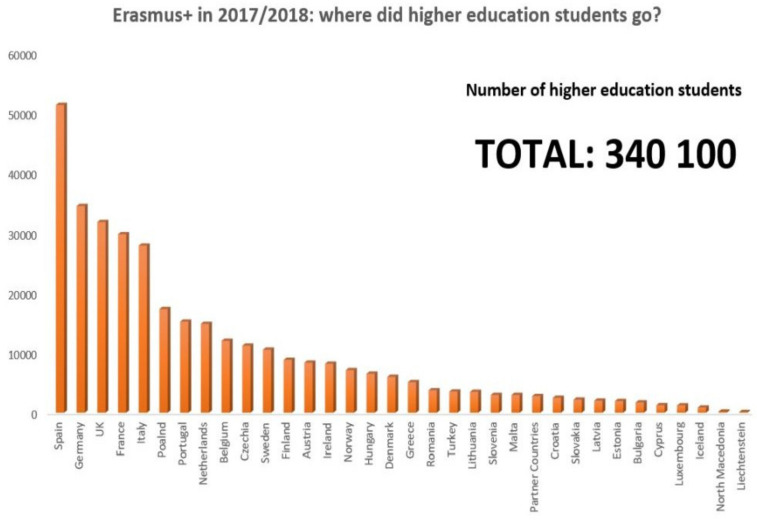
Host countries of university students with ERASMUS+ mobilities. *Source: ERASMUS Annual Report 2018,* *European Commission* [13].

Under the current ERASMUS+ program, the term study mobility was meant to describe the mobility of university students, trainees, apprentices, interns, volunteers, youth workers and staff with the aim of learning [12]. Under this new program, the flow of university students abroad in the academic year 2017–2018 can be observed in Figure 1 and Figure 2.

The concept of mobility has always been the essence of the Bologna Process, which started with the Bologna Declaration, signed in 1999. The process has become the most complex European cooperation project in the area of higher education. The best-known program is ERASMUS (European Region Action Scheme for University Student Mobility), which was set up in 1987 and supports student mobility between EU countries.

ERASMUS mobilities represent, in fact, a network between higher education institutions, whereas the nodes are the universities and countries involved, and the links are the participating students [6].

Regarding student flows and models involving ERASMUS mobility, Skribnjek [12] asserts that they fall into four separate categories: (a) studies centered on the causes of the mobility, namely push and pull factors derived from migration theory; (b) studies centered on the labor market and human capital framework so as to attract the best university students; (c) studies that highlight cross-border relations and globalization processes; and (d) studies centered on the world system theories.

In fact, ERASMUS is the world’s most successful student mobility program, reaching a total number of 4.4 million mobile university students in 2017 [13].

Among the objectives of the ERASMUS program are multilateral cooperation between educational institutions and between them and EU organizations and to increase the transparency and compatibility of the European education system. The ERASMUS program provides university students with grants to cover the additional costs of studying abroad in Europe.

As early as 2009, the education ministers of the EU set an ambitious target to be achieved by 2020, namely that a minimum of one-fifth of graduates in the European Higher Education Area should have taken a stage abroad during their study years [14]. To date, statistics show that the target was not met, though.

Nevertheless, the dynamics of mobility have been impressive. In the first year of its existence, 1987–1988, just 3244 students from 11 countries spent a study period abroad on ERASMUS. In 2013–2014, 212,208 students from 34 countries spent study time abroad. Thus, the initial goal of supporting 3 million students to study abroad was reached during the academic year 2012–2013 [15].

The budget of the ERASMUS program increased accordingly. If under the umbrella of the Lifelong Learning Program 2007–2013, ERASMUS had an allocation of almost 7 billion euro, for the new phase, called ERASMUS+, for the period 2014–2020, the budget more than doubled to 14.7 billion euro. The new goal is to reach 3.1 million university students within the ERASMUS program during 2021–2027, with an initial proposal to raise the budget to around 30 billion euro [16].

The ERASMUS program has also led to initiatives to standardize educational policies in the European Union by integrating the curriculum so that the academic achievement of the participating university students is recognized in their home institutions [5].

The mobilities contribute to the personal and professional development of students by enhancing their academic performance and giving them the chance to acquire transferable skills that are well-received by companies/organizations. Students certainly improve foreign language skills and also develop an enhanced cross-cultural empathy. They become quicker to adapt to changes and new environments, overcome emotional challenges, solve problems, participate in teamwork, think in critical terms, be tolerant and communicate better. Mobility boosts job prospects and opens the mind to different cultures [15,17].

The mobilities are bound to increase the employability of future graduates. As an impact study has revealed, the unemployment rate for ERASMUS participants is 23% lower 5 years after graduation by comparison with non-participating students [11]. Moreover, most employers appreciate that international experience is an asset for job candidates and leads to higher professional responsibility.

The ERASMUS program also brings a number of benefits to universities, such as: gaining international experience and respect, education in an intercultural environment, research contributions and funding sources for projects [18].

University students are motivated to participate in mobility programs, as shown in the literature, by employment and accommodation opportunities, value-addedf student career, social events, the deterring costs involved with international education, to gain international education and life experiences, to develop basic international skills and to improve academic performance and foreign languages [19,20,21,22,23,24,25,26].

Previous studies show that when students choose a university to attend in the ERASMUS program, they consider professional aspects, personal preferences and financial issues [27]. The choice of the country is made taking into account the number of top universities and those in which their colleagues have been before [3,28].

The most important challenges toward mobilities differ slightly between outgoing and incoming university students. According to an EU-wide survey by the Bologna Follow-Up Group, for outward mobilities, funding is the dominant concern, followed by bureaucratic and organizational difficulties, students’ personal emotional challenges, such as leaving family, friends and workplace, and difficulties of recognition of mobility periods. For incoming mobilities, the lack of support services and accommodation tops the list, followed by funding, curriculum and study organization, a lack of information, behavioral problems, as well as insufficient knowledge of the language [10,29,30].

The COVID-19 pandemic has changed the dynamics of the whole world, including the higher education area. Students, teachers and administration alike have faced new challenges never seen before. The pandemic has also modified the weight of factors that determine students’ decisions of studying abroad and choosing a respective country [31]. The activities at universities needed to be revised to comply with sanitary conditions imposed by national authorities. According to the degree of autonomy, each higher education institution has established its new working conditions.

The most severe challenges for ERASMUS students arose from the quarantine measures for getting in and out of a particular country [32]. Moreover, in the countries where lockdown was imposed, universities were forced to shut down and move activities online. ERASMUS mobilities were thus negatively affected by the pandemic [33] and had to be adapted to the new conditions [34]. According to IOM (2021), around 180 countries have adopted 755 exceptions to COVID-19 rules during the pandemic, opening up for mobilities despite travel restrictions, while 11 countries have eliminated 15 exceptions [35]. The students who were already participating in these mobilities were isolated from their families and their home countries. Some of those who were preparing to leave needed to cancel their mobility. As observed by Stewart and Lowenthal (2021), the move to online learning has placed ERASMUS students in a special, uncomfortable position compared to fellow students who were studying from home [36].

As stated by Marinoni et al. (2020), due to the COVID-19 pandemic, 60% of universities have adopted virtual mobilities or online learning to substitute for the physical mobilities of students [37]. Tereseviciene et al. (2011) have defined virtual mobilities as a form of learning and communication based on the cooperation of at least two higher education institutions coming from different backgrounds and cultures; a virtual element of the learning environment supported by information technology; and the knowledge exchange hereby improving cross-cultural competences [38]. Compared to the physical ones, virtual mobilities crossed space constraints and have played a vital part in keeping student exchange afloat in the context of travel restrictions during the pandemic [31].

However, the recent study by Anas et al. (2022) on the effects of the COVID-19 pandemic on students participating in mobilities shows that students lacked the physical environment and social interaction, as well as the competencies and intercultural skills enjoyed in a typical academic framework [33]. Besides the educational activities pegged to the mobilities, the pandemic has also affected local economies and revenues of cities used to incoming students, with figures falling significantly, which has led to a decline in the real estate market, consumption and in the tourism industry [39].

## 3. Materials and Research Methods

### 3.1. The Research Context

**Figure 3:**
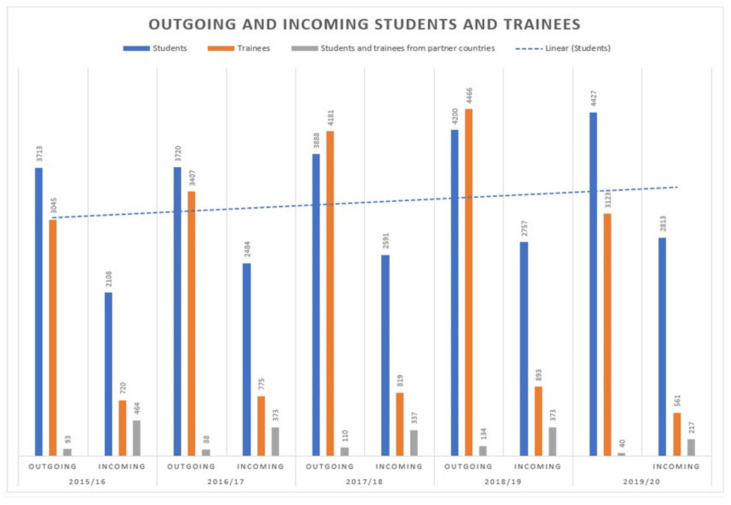
ERASMUS+ student flows to and from Romania. *Source: Adapted by the authors from data published in ERASMUS+ Annual Report Factsheets Romania, European Commission* [40].

Over 7500 university students from Romania go on ERASMUS mobility every year. At the same time, Romania is chosen as a destination by approximately 3500 students from Europe and the rest of the world. Figure 3 shows the recent evolution of Romanian outgoing and foreign incoming student numbers under the ERASMUS program.

At the Transilvania University of Brasov (UNITBV) and the Faculty of Economic Sciences and Business Administration (SEAA), the flow of students with ERASMUS mobility has evolved, as can be seen in Table 1.

The data show that the number of Romanian university students leaving with ERASMUS mobility varied during this period by around 128 per year, and the number of students from the Faculty of Economic Sciences and Business Administration (the largest faculty) leaving with ERASMUS scholarships varied by around 22 students per year. It can be observed that the number of university students leaving through the ERASMUS mobility program has decreased slightly in the last 3 years (the slump in the last year is explainable due to the pandemic).

The destination countries preferred by the students from the Transilvania University of Brasov included in the sample of the research were France, Belgium, Spain and the United Kingdom (Table 2).

### 3.2. Research Methodology

The aim of the research was to obtain feedback from university students who participated in the ERASMUS program, to analyze the benefits and challenges of participating in this program and to identify the reasons that led students to choose a particular partner university. The research conducted is a quantitative one of opinion poll type.

The statistical population is composed of the 1059 outgoing students from the Transilvania University of Brasov who have benefitted from ERASMUS mobilities during the period 2012–2021 (Table 1). Transilvania University is the largest higher education institution in the center of Romania and one of the largest in the country, ranking fourth for outgoing students inside the ERASMUS program. Based on these premises, it is fairly reasonable to accept that the sample chosen is representative of the behavior of Romanian students at the national level.

Given the theme of the paper, the research was based on the following specific objectives:

- Identifying the main reasons that determine Romanian students from Transilvania University to choose a certain partner university;
- Identifying the countries that Romanian students from Transilvania University choose to carry out their ERASMUS mobility;
- Determining the main benefits of participating in the ERASMUS program;
- Determining the main challenges encountered by students from Transilvania University in the ERASMUS program.

The opinion polling technique was employed by means of a computer-administered questionnaire as a data collection tool, using the method called Computer-Assisted Web Interviewing (CAWI) [41]. Due to the empirical nature of the research, a non-probabilistic sampling method was used, namely the “snowball” type. In practice, only university students who meet the criteria for inclusion in the research, students who have left with ERASMUS mobility from Transilvania University, were considered.

The final sample was composed of 112 outgoing students. Generally, the standard error indicates the representativeness of the sample. In the above context, the calculated standard error is 5%, with a confidence level of 95%. Taking into account the statistical population of 1059 persons, the size of the sample should have been larger. However, this is preliminary research. Based on the results obtained from the present research, another grounded, more ample study will be performed in the future, with an extended sample to comprise students from more Romanian and possibly European universities.

After collecting the information with the help of the questionnaire, the statistical data were processed with the SPSS system (Statistical Package for Social Sciences).

Given the exploratory nature of the research, the aim was to obtain as many questionnaires as possible completed by students at Transilvania University. The study was conducted on a sample of 112 university students, structured as follows: 76% female, 24% male. Of these, 63% completed mobility during the Bachelor’s cycle, and 37% during the Master’s degree. The age of the respondents is shown in Table 1. It is observed that the majority of respondents (50%) are between 25 and 30 years old, followed by those aged between 21 and 25 (39.3%) (Table 3).

## 4. Results

The first part of the study examines the behavioral reasons behind the choice of mobility abroad, such as: the reputation of the university through its teaching and research activities, the attractiveness of that respective country, costs involved, employment opportunities and relatives and friends living in that country; all of them taking into account the funding provided by the ERASMUS program and the additional revenues and resources.

To study these reasons, we used the analysis of multiple dichotomous responses. Following the application of this analysis, there were no missing data on the variable providing reasons for participation in ERASMUS and no gender differences in reasons for participation were found.

Identifying the key emotional factors that lead university students to choose ERASMUS mobility, as well as improving mobility conditions, is essential for creating educational policies, regulations and guidelines that will attract as many students as possible to such programs. Such experiences are beneficial for society, the economy, professional development, academic performance and improvement of students’ skills and for the development of empathic international behavior, bound to foster cooperation.

One of the important aspects to be analyzed inside the topic of international scholarships is the emotional behavior that drives university students to pursue a study period in a foreign country. When investigating the reasons for which students chose to participate in a study mobility abroad, most of the respondents (68.8%), especially Master’s students, indicated the desire to study in another country. The second most important rationale was composed of the characteristics of the host country itself and the educational offer of the host university (58% each).

As an interesting finding, the reputation of the host university was deemed two times higher by Master’s students compared to Bachelor’s students, a fact that is related to the higher maturity in their educational career. The study shows that a very small percentage of individual respondents chose to study abroad to be closer to their family and relatives in that country, only 8% (Table 4).

These results are in line with those of other studies [18,42], which show that university students want to study abroad for new experiences, to have better access to academic information, and to improve their foreign language skills. Ekti [18] found that the academic grading average scores of 10 out of 40 students were higher in the university where they left with the ERASMUS program than before going abroad for studies, which confirms that students want to go abroad for study in other countries to improve their academic performance in the chosen field and that they consciously take full advantage of the ERASMUS program.

Naturally, when embarking on a study period abroad for one semester or two, expectations mount in the minds of the scholarship holders. The most cited desires related to their mobility in a foreign country were to gather useful information and improve academic performance for their future career, as well as visiting new places and getting to know another culture (91.1% of answers in each case). These answers were evenly spread between male and female students.

About half of the respondents have also indicated that learning a foreign language and making new friends were among the expectations they had when starting their study mobilities abroad, with a slightly higher frequency for male students (Table 5). These results can be correlated with the fact that as they mature, students better understand the need for and importance of knowing several languages.

In the process of getting accustomed to studying abroad, students stumble upon various emotional and behavioral challenges during their stay in the foreign country. When asked about what the major challenges they have faced, respondents indicated that they found a major difficulty in adapting to the teaching and examination methods (46.5% of cases), adapting to the work/academic style in the host university (35.4% of cases), insufficient knowledge of the foreign language (23.2% of cases), getting accustomed to the local culture (15.2% of cases), and poor communication with colleagues (13.1% of cases).

When comparing the answers on the two levels of study, Master’s students had greater difficulty in communicating with colleagues than their undergraduate colleagues (empathy), while Bachelor’s students found it more difficult to adapt to the university work style (behavioral problems); both categories having approximately the same level of adaptation to the teaching and assessment methodology. However, Bachelor’s students have found it harder to cope with low language skills and empathize with the particular culture when compared to Master’s students (Table 6).

Similar results can be found in the study of Pappa et al., 2013 [43], which shows that among the behavioral problems faced by students attending partner universities through the ERASMUS program were: support for finding accommodation, misunderstanding of the credit transfer system (ECTS), differences in teaching methods, poor student–teacher relationship and administrative difficulties.

In order for the study mobility to be acknowledged as a success by the participant, a group of factors related to the host university needs to be in place and properly addressed. The majority of respondents considered that the educational component itself, the way teaching was done, including the student–teacher relationship, was the most fulfilling aspect of mobility (90.2% of cases). Further, the relationship with academic services (69.6% of cases), the organization of events, shows, fairs by the host university (64.3% of cases), as well as the organization of trips for students (63.4% of cases) were also highly appreciated. Fewer than half of the students have shown positive appraisal for accommodation conditions (43.8% of cases) and the canteen services (40.2% of cases). With a few exceptions, answers did not differ widely between university students from the two academic levels—Bachelor’s and Master’s (Table 7).

As also shown by Endes [44], university students who went to study in other countries appreciated the accessibility of academic counselors and teachers, teaching methods, library and information resources.

When asked about the services related to the host university that caused emotional distress to students undertaking mobilities, two major aspects were revealed. On the one side, Bachelor’s students ranked canteen/food on the first spot of the negative rated aspects with a total of 20% of responses. Master’s students, on the other side, ranked accommodation services in the first place in their negative hierarchy, with a total of 18.8% of responses. Apart from these two components, there were no other notable complaints (Table 8).

An important goal of any student pursuing mobility abroad is the benefit they try to reap for their future career. Taking into consideration that the research was undertaken among graduates, the authors were able to approach the issue of the relationship between the ERASMUS stay abroad and jobs afterward. The result leaves no doubt for interpretation. The vast majority of respondents (89%) have asserted that their mobility in a foreign country has helped them in their future professional careers.

There was a significant difference between the observed frequencies and those expected for the participants in the Bachelor’s and Master’s degree cycle and the perception of a benefit as a result of participating in the ERASMUS mobility program. This difference was analyzed by applying the association’s Chi-Square Test (Chi-Square of independence).

Table 9 indicates the observed and expected frequencies of the cases and the difference (residual value) between them for each cell. The observed frequency (called “Count”) is shown first, then the expected frequency (called “Expected Count”). The observed frequencies are always integers so that they are easy to locate.

The Expected Count line features the estimated frequencies. From the summary analysis of the differences between the absolute frequencies (Count) and the estimated ones (Expected Count), particular differences can be identified at the level of all subgroups. The final column in Table 9, labeled “Total”, contains the number of cases in that row, followed by the expected number of cases in the table. Chi-Square Tests (Table 10) are used to test the significance of the differences.

By applying this test, whose result values are Pearson Chi-Square = 0.647, df(1) and *p* > 0.05, we can state that there is not enough evidence to reject the null hypothesis of no association between the educational cycle and the benefits of participating in ERASMUS mobility.

Although one might think that Master’s students are more mature and perceive the benefits that such a scholarship brings in a better way, the results show that this does not happen in real terms.

This means that these mobilities need to be continued as part of the educational policies and even extended to facilitate the exchange of information, culture, etc., between university students from different countries. This is also highlighted by Bryla [45] in his study, which showed that one-third of ERASMUS students considered that international experience has a significant influence on their personal fulfillment, their academic performance and their professional position. Almost one-fifth of the respondents indicated ERASMUS mobility as a paramount factor associated with these achievements.

According to Bryla [45], only 1.6% of the surveyed university students who participated in ERASMUS mobilities mentioned that they had never had jobs (compared to 2.5% in the control group). Of those who had a job, 51% had a permanent employment contract, 28% had a fixed-term contract and 21% had a different type of contract. The average period for fixed-term contracts was of almost two years, and the median one was of one year. The same author points out that most of the former participants in ERASMUS mobilities were employed in organizations with international activity, which proves that these students have a better command/knowledge of foreign languages and are more attractive to these companies.

Another study [46] highlights the impact of the ERASMUS mobility on the professional and scientific fulfillment of exchange students. Influence has occurred in the following areas: acquiring scientific and professional knowledge as well as academic performance, acquiring foreign language skills, identifying academic, scientific and professional opportunities, studying several aspects of students’ emotional behavior, developing empathy, social skills and a personal system of values, displaying self-confidence and building a state of independence.

For a long period of time, university students have complained that the financial grant from the European Union was not enough to cover all necessary costs during their stay abroad so they were determined to find an alternative source of funding. The results of the research confirm this situation, with most of the respondents (90%) stating that they have used a supplementary funding source to manage all the incurred costs during the mobility in another university.

Applying the Chi-square test (Table 11) for the degree of correspondence between the observed and the expected frequencies, it is shown that while the frequency of those who consider that the scholarship covers more than 75% of the monthly expenses does not differ much from the theoretical frequency, in the case of other answers, the observed frequency differs significantly from the theoretical one.

Thus, those who consider that the scholarship covers less than 25% of the monthly expenses are very few, and those who consider that the scholarship covers 51–75% of the monthly expenses are the most.

These results correlate with previous studies, which show that for students who go on an ERASMUS scholarship to a foreign university, the main challenge is the amount of the scholarship, which does not fully cover the expenses related to the stay in a foreign country [10,29,30]. The results of the Chi-square test (46.83), df(4) and *p* < 0.001 justify the conclusion that the differences between the five types of responses are significant and are not due to the random variation of sampling.

As a consequence of not being able to cover all the typical costs that appeared during the ERASMUS stay abroad, most of the respondents (64% of cases) asserted that some supplementary financial benefits related to the scholarship would be really helpful.

One of the important features that influences the decision for one destination or another is the way universities promote themselves to future candidates. Promotion takes place by means of a diversity of channels. In the view of former ERASMUS university students, the best method for promotion is to use social media networks (90% of cases) and the own website of the university (87% of cases). Another possibility would be to participate in educational fairs (65% of cases) and much less through the use of leaflets and brochures (24% of cases) or radio channels (18% of cases). There were no significant deviations between the answers of respondents from the two academic levels, with the exception of radio as a means of promotion, which was favored by Master’s students twice as much as by Bachelor’s students. These answers are related to the fact that learners are familiar and make extensive use of social networks and websites, generally online, for information.

## 5. Discussion

The ERASMUS program is one of the most important international exchange programs for students. More than three million participants have already enjoyed this experience, with female students being more actively represented compared to male students [47]. ERASMUS is proving its value to those taking part in it. A total of 95% of university students say they are “satisfied” or “very satisfied” with their experience. Furthermore, 90% of students feel emotionally more confident and ready to take on new challenges thanks to ERASMUS.

The mobility experience does not only empower people with academic performance but also new skills needed in the labor market (as previously shown by Parey and Waldinger, 2011) and learning outcomes specific to their field of study [48]. A total of 9 out of 10 ERASMUS participants improve their foreign language skills during their time abroad while also becoming more tolerant, empathic, open-minded and resilient [49].

In the context of the COVID-19 pandemic, due to limited transport possibilities and the closure of some countries, ERASMUS+ mobilities had to be reoriented from the actual mobility (movement of students to the host country and physical participation in courses) to virtual spaces. In order to take place in a regulated framework and support the move toward the digital transformation of education in Europe, it was necessary to underline more precisely the terms used in relation to the achievement of mobility.

Two new forms of mobility have been defined, virtual mobility and combined mobility. Virtual mobility refers to the period in which a participant is in the country of origin and attends online learning activities offered by the institution/organization in the host country. For the “mobility” period in a virtual format, the participant does not receive a subsistence grant or transport from EU ERASMUS+ funds. Blended mobility refers to a learning activity that is achieved by combining a period of “mobility” in virtual format and the period of mobility itself in physical format. The possibility of organizing a combined mobility implies the planning from the very beginning of a period of mobility in virtual format obligatorily correlated with a period of mobility in physical format.

The COVID-19 pandemic has shown that when conditions change dramatically, new ways of achieving these mobilities can be found so that student participation is not affected. Besides experimenting now with various forms of mobilities, such as virtual and blended mobilities, the European Union also fights against climate change and strives to reduce the carbon footprint of traditional student mobilities abroad by altering the transportation mode and offering incentives for “green travel” (without using an airplane). However, studies show that although student mobilities have increased over the years, they did not necessarily entail a rise in carbon emissions as the typical pattern leans toward regionalization [50].

The above novelties in the way the ERASMUS program operates are included in a package of priorities. At the core of this package stands an inclusion and diversity strategy, where special attention is provided to students with fewer opportunities, stemming from minorities or low-income families, persons with refugee status, students with disabilities or severe health problems, so as to remove all potential barriers to mobility [51].

## 6. Conclusions

In today’s competitive academic environment, the choice of students concerning the future host university where they will spend a study period abroad takes into account various factors. Some interesting findings emerged from the present research, such as the fact that students rank their potential increased academic performance on an equal level with the social aspect of getting to know other cultures. Moreover, emotional challenges differ between Bachelor’s students (low language skills, complaints about canteen) and Master’s students (poor communication with colleagues, uncomfortable accommodation). While students generally found it hard to cope with new teaching and examination methods, they ranked the relationship with the teachers as the major success factor of the mobility.

The main limit of this research is given by the impossibility of extending the obtained results, a limit imposed by the type of the research method used and its empirical nature. Only the behavioral perceptions of mobile students from a single university from a single country (Romania) were identified and investigated in this research. Despite these limitations, the authors consider that the paper brings valuable empirical contributions to analyzing the most important issues and emotional challenges of student mobilities abroad. It also highlights the areas in which universities can improve the efficiency of mobilities for all participants and apply modern educational measures. Such measures will be stringently needed as competition between universities has intensified due to the COVID-19 pandemic, so higher education institutions search for viable business models to attract foreign students.

Starting from these premises, future research can be extended in order to assess the behavioral perceptions of students from other universities in EU member states. This would allow the extension of the findings, paving the way for comparative research across universities in the EU. Given the longstanding cooperation inside the ERASMUS network of the Transilvania University of Brasov and the experience gathered herein, the authors consider teaming up with researchers from partner universities, especially from major student-outbound countries (France, Germany, Spain, Italy) to test the same approach with multicultural samples and to compare results for a European-wide perspective, in a framework based on the ERASMUS priorities. As such, research on educational mobilities and the emotional traits of participating students would bring significant opportunities on behalf of EU policymakers when designing the educational policies and guidelines for future programs.

## Figures and Tables

**Table 1:** The evolution of the outgoing study mobility of students of the Transilvania University of Brasov through the ERASMUS program in the period 2012–2021.

Academic Year	No. of UNITBV Students	No. of SEAA Students
2012–2013	133	22
2013–2014	122	24
2014–2015	134	32
2015–2016	129	20
2016–2017	132	19
2017–2018	126	20
2018–2019	120	20
2019–2020	117	28
2020–2021	46	13

*Source: Data processed by the authors from internal documents.*

**Table 2:** Countries where ERASMUS students from the Transilvania University of Brasov completed their mobility.

	Female	Male	Frequency	Percent	Valid Percent	Cumulative Percent
Austria	1	1	2	1.8	1.8	1.8
Belgium	16	2	18	16.1	16.1	17.9
Czechia	6	2	8	7.1	7.1	25.0
Croatia	2	1	3	2.7	2.7	27.7
Denmark	0	1	1	0.9	0.9	28.6
Finland	6	1	7	6.2	6.2	34.8
France	14	5	19	17.0	17.0	51.8
Germany	5	2	7	6.2	6.2	58.0
Greece	2	2	4	3.6	3.6	61.6
Ireland	5	1	6	5.3	5.3	66.9
Italy	3	1	4	3.6	3.6	70.5
England	9	2	11	9.8	9.8	80.3
Netherlands	1	1	2	1.8	1.8	82.1
Poland	2	0	2	1.8	1.8	83.9
Scotland	0	1	1	0.9	0.9	84.8
Slovakia	0	2	2	1.8	1.8	86.6
Spain	13	0	13	11.6	11.6	98.2
Turkey	0	1	1	0.9	0.9	99.1
Hungary	1	0	1	0.9	0.9	100.0
**Total**			**112**	**100.0**	**100.0**	

*Source: Authors’ own research.*

**Table 3:** Age of the surveyed respondents.

		Frequency	Percent	Valid Percent	Cumulative Percent
Valid	21–25 years	44	39.3	39.3	39.3
	25–30 years	56	50.0	50.0	89.3
	Over 30 years	6	5.4	5.4	94.6
	Under 21 years	6	5.4	5.4	100.0
	**Total**	**112**	**100.0**	**100.0**	

*Source: Authors’ own research.*

**Table 4:** Reasons for choosing mobility.

	Study Cycle		Total Responses	Percent of Cases
	Bachelor	Master		
Country	14.6%	10.3%	24.9%	58%
University reputation	2.7%	5%	7.7%	17.9%
Educational offer	14.9%	10%	24.9%	58%
Costs	2.7%	2.7%	5.4%	12.5%
Relatives/friends in the host country	2.3%	1.1%	3.4%	8%
Desire to study in another country	16.4%	13.1%	29.5%	68.8%
Other	2.7%	1.5%	4.2%	9.8%
**Total**	**56.3%**	**43.7%**	**100%**	**233%**

*Source: Authors’ own research.*

**Table 5:** Expectations related to the mobility in which they participated.

	Gender		Total Responses	Percent of Cases
	Male	Female		
Gather information and improve academic performance useful for my professional career	24.9%	7.3%	32.2%	91.1%
Learn a new language	14.2%	3.5%	17.7%	50.0%
Make new friends	13.2%	4.8%	18.0%	50.9%
Visit new places and get to know other cultures	24.3%	7.8%	32.1%	91.1%
**Total**	**76.6%**	**23.4%**	**100.0%**	**283.0%**

*Source: Authors’ own research.*

**Table 6:** Challenges encountered during the mobility.

	Study Cycle		Total Responses	Percent of Cases
	Bachelor	Master		
Insufficient knowledge of the language	11.3%	3.9%	15.2%	23.2%
Empathizing with the local culture	7.3%	2.6%	9.9%	15.2%
Adapting to the university work style	15.2%	8%	23.2%	35.4%
Adapting to the teaching and assessment methods	15.9%	14.6%	30.5%	46.5%
Poor communication with colleagues	3.3%	5.3%	8.6%	13.1%
Other	8.6%	4%	12.6%	19.2%
**Total**	**61.6%**	**38.4%**	**100%**	**152.5%**

*Source: Authors’ own research.*

**Table 7:** Services that were appraised at the host university.

	Study Cycle		Total Responses	Percent of Cases
	Bachelor	Master		
The educational act itself	12.9%	8.8%	21.7%	90.2%
Accommodation conditions	7.5%	3%	10.5%	43.8%
Canteen/Food	4.9%	4.7%	9.6%	40.2%
Communication with administrative services	10.5%	6.2%	16.7%	69.6%
Organizing shows and events	9.2%	6.2%	15.4%	64.3%
Organizing trips	9.4%	5.8%	15.2%	63.4%
Other extracurricular activities	6.8%	4.1%	10.9%	45.5%
**Total**	**61.2%**	**38.8%**	**100%**	**417.0%**

*Source: Authors’ own research.*

**Table 8:** Services that arose emotional distress at the host university.

	Study Cycle		Total Responses	Percent of Cases
	Bachelor	Master		
The educational act itself	5%	2.5%	7.5%	9.1%
Accommodation conditions	11.2%	18.8%	30.0%	36.4%
Canteen/Food	20%	8.7%	28.7%	34.8%
Communication with administrative services	7.5%	8.8%	16.3%	19.7%
Organizing shows and events	5%	1.2%	6.2%	7.6%
Organizing trips	3.8%	7.5%	11.3%	13.6%
**Total**	**52.5%**	**47.5%**	**100%**	**121.2%**

*Source: Authors’ own research.*

**Table 9:** Observed and expected frequencies.

			Study Cycle		Total
			Bachelor	Master	
Mobility benefits	**Yes**	Count	62	38	100
		Expected Count	60.7	39.3	100.0
	**No**	Count	6	6	12
		Expected Count	7.3	4.7	12.0
**Total**		Count	68	68	44
		Expected Count	68.0	68.0	44.0

*Source: Authors’ calculations based on collected data.*

**Table 10:** Critical report for Chi-Square analysis.

	Value	df	Asymptotic Significance (2-Sided)	Exact Sig. (2-Sided)	Exact Sig. (1-Sided)
Pearson Chi-Square	0.647 ^a^	1	0.421		
Continuity Correction ^b^	0.242	1	0.623		
Likelihood Ratio	0.634	1	0.426		
Fisher’s Exact Test				0.534	0.307
N of Valid Cases	112				

*^a^ Computed only for a 2 × 2 table. ^b^ 0 cells (,0%) have expected count less than 5. The minimum expected count is 212.13. Source: Authors’ calculations based on collected data.*

**Table 11:** Observed frequencies, expected frequencies and residual values.

	Observed N	Expected N	Residual
Under 25%	3	22.4	−19.4
Between 26–50%	32	22.4	9.6
Between 51–75%	43	22.4	20.6
Over 75%	24	22.4	1.6
100%	10	22.4	−12.4
**Total**	**112**		

*Source: Authors’ calculations based on collected data.*

## Data Availability

The data presented in this study are available on request from the corresponding author. The data are not publicly available due to privacy reasons.
